# Supplementary material for: Association of Exposure to Phthalate Metabolites with Antenatal Depression in US Pregnant Women
Source: Toxics. 2025 Sep 30;13(10):838. doi: 10.3390/toxics13100838 (PMC12568236; doi:10.3390/toxics13100838)
Supplement: Supplementary file 1 [file toxics-13-00838-s001.zip › toxics-3830080-supplementary.pdf]

*Article*

# Association of Exposure to Phthalate Metabolites with Antenatal Depression in US Pregnant Women

**Pallavi Dubey *, Chinthana Thangavel, Abdelrahman Yousif, Sophie Kim and Sireesha Reddy ***

Department of Obstetrics and Gynecology, Paul L. Foster School of Medicine, Texas Tech University Health Sciences Center El Paso, El Paso, TX 79905, USA; cthangav@ttuhsc.edu (C.T.); yousif.abdelrahman@ttuhsc.edu (A.Y.); kim72939@ttuhsc.edu (S.K.)
* Correspondence: paldubey@ttuhsc.edu (P.D.); sireesha.reddy@ttuhsc.edu (S.R.)

**Supplementary Table S1.** Comparisons of phthalate concentrations according to depression status

| **Phthalates** | No depression (PHQ-9 Score≤4) | Mild to severe depression (PHQ-9 Score>4) | p-value |
|---|---|---|---|
| N | 143 | 65 | |
| Mono(carboxynonyl) phthalate (ng/mL), median (IQR) | 1.60 (0.81, 3.00) | 2.40 (1.50, 4.50) | 0.002 |
| Mono(carboxyoctyl) phthalate(ng/mL), median (IQR) | 3.90 (2.20, 10.10) | 6.40 (3.50, 14.60) | 0.010 |
| Mono-2-ethyl-5-carboxypentyl phthalate (ng/mL), median (IQR) | 14.30 (6.00, 25.90) | 21.00 (10.68, 38.80) | 0.017 |
| Mono-n-butyl phthalate (ng/mL), median (IQR) | 11.26 (5.60, 26.10) | 21.40 (11.67, 42.40) | <0.001 |
| Mono-(3-carboxypropyl) phthalate (ng/mL), median (IQR) | 1.30 (0.60, 2.40) | 2.10 (1.16, 4.30) | <0.001 |
| Mono-ethyl phthalate (ng/mL), median (IQR) | 50.49 (19.73, 210.60) | 115.70 (41.12, 254.30) | 0.010 |
| Mono-(2-ethyl-5-hydroxyhexyl) phthalate (ng/mL), median (IQR) | 9.10 (3.30, 18.52) | 14.00 (6.10, 24.20) | 0.011 |
| Mono-(2-ethyl)-hexyl phthalate (ng/mL), median (IQR) | 1.60 (0.85, 3.60) | 3.20 (1.24, 6.70) | 0.006 |
| Mono-isobutyl phthalate (ng/mL), median (IQR) | 4.90 (1.90, 9.50) | 9.71 (6.30, 16.80) | <0.001 |
| Mono-isononyl phthalate (ng/mL), median (IQR) | 0.87 (0.87, 0.87) | 0.87 (0.64, 0.87) | 0.370 |
| Mono-(2-ethyl-5-oxohexyl) phthalate (ng/mL), median (IQR) | 7.10 (2.90, 13.90) | 11.20 (6.60, 22.90) | 0.007 |
| Mono-benzyl phthalate (ng/mL), median (IQR) | 5.33 (2.02, 12.00) | 12.10 (4.74, 22.90) | <0.001 |

Abbreviations: IQR, Inter quartile Range; PHQ, Patient Health Questionnaire.

**Supplementary Table S2.** Adjusted association of a high level of each phthalate metabolite with depression scores

| **Phthalates** | **aRC* (95% CI)** | **p-value** |
|---|---|---|
| High mono (carboxynonyl) phthalate (ng/mL) | 0.19 (-0.04, 0.43) | 0.106 |
| High mono (carboxyoctyl) phthalate (ng/mL) | 0.12 (-0.13, 0.37) | 0.334 |
| High mono-2-ethyl-5-carboxypentyl phthalate (ng/mL) | 0.27 (0.03, 0.5) | 0.026 |
| High Mono-n-butyl phthalate (ng/mL) | 0.40 (0.17, 0.63) | 0.001 |
| High Mono-(3-carboxypropyl) phthalate (ng/mL) | 0.30 (0.07, 0.53) | 0.011 |
| High Mono-ethyl phthalate (ng/mL) | 0.27 (0.05, 0.5) | 0.019 |
| High Mono-(2-ethyl-5-hydroxyhexyl) phthalate (ng/mL) | 0.30 (0.07, 0.54) | 0.010 |
| High Mono-(2-ethyl)-hexyl phthalate (ng/mL) | 0.32 (0.08, 0.55) | 0.008 |
| High Mono-isobutyl phthalate (ng/mL) | 0.33 (0.08, 0.58) | 0.009 |
| High Mono-isononyl phthalate (ng/mL) | -0.02 (-0.29, 0.25) | 0.887 |
| High Mono-(2-ethyl-5-oxohexyl) phthalate (ng/mL) | 0.34 (0.12, 0.57) | 0.003 |
| High Mono-benzyl phthalate (ng/mL) | 0.39 (0.16, 0.63) | 0.001 |

Abbreviations: aRC, Adjusted Regression Coefficient. Log-transformed values of depression scores and phthalate concentrations were included in the analyses. *Analyses were adjusted for age, ethnicity/race, education, marital, income status, smoking, alcohol use, physical activity and obesity.

**Supplementary Table S3.** Unadjusted association of a high level of each phthalate metabolite with depression levels.

| Phthalate metabolites | OR* (95% CI) | p-value |
|---|---|---|
| High mono (carboxynonyl) phthalate (ng/mL) | 1.92 (1.06, 3.48) | 0.032 |
| High Mono (carboxyoctyl) phthalate (ng/mL) | 2.02 (1.09, 3.73) | 0.025 |
| High Mono-2-ethyl-5-carboxypentyl phthalate (ng/mL) | 1.99 (1.1, 3.6) | 0.024 |
| High Mono-n-butyl phthalate (ng/mL) | 3.67 (1.97, 6.83) | <0.001 |
| High Mono-(3-carboxypropyl) phthalate (ng/mL) | 2.24 (1.23, 4.08) | 0.008 |
| High Mono-ethyl phthalate (ng/mL) | 2.34 (1.29, 4.28) | 0.005 |
| High Mono-(2-ethyl-5-hydroxyhexyl) phthalate (ng/mL) | 2.18 (1.2, 3.95) | 0.010 |
| High Mono-(2-ethyl)-hexyl phthalate (ng/mL) | 2.6 (1.42, 4.77) | 0.002 |
| High Mono-isobutyl phthalate (ng/mL) | 3.14 (1.7, 5.81) | <0.001 |
| High Mono-isononyl phthalate (ng/mL) | 1.08 (0.54, 2.18) | 0.822 |
| High Mono-(2-ethyl-5-oxohexyl) phthalate (ng/mL) | 2.35 (1.27, 4.32) | 0.006 |
| High Mono-benzyl phthalate (ng/mL) | 3.04 (1.64, 5.61) | <0.001 |

Abbreviations: PHQ, Patient Health Questionnaire; OR, Odds Ratio. *Analyses were adjusted for age, ethnicity/race, education, marital, income status, smoking, alcohol use, physical activity and obesity.

**Supplementary Table S4.** Relative importance of combination of phthalate metabolites obtained using weighted quantile sum (WQS) regression analysis

| Phthalates | Weight |
|---|---|
| Mono (carboxynonyl) phthalate (ng/mL) | 0.317 |
| Mono-benzyl phthalate (ng/mL) | 0.309 |
| Mono-isobutyl phthalate (ng/mL) | 0.119 |
| Mono-n-butyl phthalate (ng/mL) | 0.083 |
| Mono-(2-ethyl)-hexyl phthalate (ng/mL) | 0.066 |
| Mono-ethyl phthalate (ng/mL) | 0.044 |
| Mono-(3-carboxypropyl) phthalate (ng/mL) | 0.024 |
| Mono-isononyl phthalate (ng/mL) | 0.022 |
| Mono-(2-ethyl-5-hydroxyhexyl) phthalate (ng/mL) | 0.011 |
| Mono-(2-ethyl-5-oxohexyl) phthalate (ng/mL) | 0.003 |
| Mono-2-ethyl-5-carboxypentyl phthalate (ng/mL) | 0.002 |
| Mono (carboxyoctyl) phthalate(ng/mL) | 0.001 |

Log-transformed values of depression scores and phthalate concentrations were included in the analyses.
